# Antimicrobial Activity of Calcium Hydroxide and Betamethasone on Enterococcus *faecalis*; An *in vitro* Assessment

**DOI:** 10.7508/iej.2015.03.008

**Published:** 2015-07-01

**Authors:** Mahdi Tabrizizadeh, Mojtaba Rasti, Fatemeh Ayatollahi, Mohammad Hossein Mossadegh, Hengameh Zandi, Farzad Dehghan, Zohreh Mousavi

**Affiliations:** a *Department of Endodontics, Dental School, Shahid Sadoughi University of Medical Sciences, Yazd, Iran; *; b * Private Practice, Yazd, Iran; *; c * Department of Pharmacology, Shahid Sadoughi University of Medical Sciences, Yazd, Iran; *; d * Department of Pathobiology, Shahid Sadoughi University of Medical Sciences, Yazd, Iran;*; e * Dental School, Shahid Sadoughi University of Medical Sciences, Yazd, Iran; *; f * Medical Information and Library Science, Dental School, Shahid Sadoughi University of Medical Sciences, Yazd, Iran*

**Keywords:** Antimicrobial Activity, Betamethasone, Calcium Hydroxide, *Enterococcus faecalis*, Intracanal Medication

## Abstract

**Introduction:**

Calcium hydroxide (CH) is one of the most common intracanal medications. Corticosteroids (CS) are used in endodontics because of their anti-inflammatory activity. This study aimed to evaluate the antimicrobial effect of CH+betamethasone and CH+saline against *Enterococcus faecalis* (*E. faecalis*) using agar diffusion test and measuring the microbial zone of inhibition (ZOI).

**Methods and Materials:**

Four plates containing Mueller-Hinton broth and *E. faecalis* culture media, were prepared. In each plate, 5 holes (5×3 mm) were created and a creamy mixture of CH+betamethasone was inserted into the holes (10 holes for each material). Two holes with ampicillin disks and two empty holes were used as negative and positive controls, respectively. Plates were incubated for 24 h and then the diameter of microbial ZOI was measured. The pH of each mixture was measured by pH meter. Data were analyzed using the Mann-Whitney U test.

**Results:**

The mean diameter of ZOI for CH+betamethasone and CH+saline was 3.4 and 3 mm, respectively. The difference was not significant (*P*=0.143). The pH was 12.5 for CH+saline and 12.3 CH+betamethasone, respectively.

**Conclusion:**

The mixture of CH+betamethasone had good antimicrobial effects against *E. faecalis*. Further studies are needed to confirm the value of this mixture in clinical settings.

## Introduction

إacteria and their by-products are the main causes of pulp and periapical inflammation. So elimination or reducing the bacterial load is the main purpose of endodontic treatment [1, 2]. Mechanical cleaning of the root canal individually can’t disinfect the canal precisely, because the bacteria can hide in out-of-reach areas, isthmi, dentinal tubules and apical delta [3]. In such cases, use of intra-canal medication with antibacterial agents can be helpful. Calcium hydroxide (CH) is one of the most popular intra-canal medications [4]. Although the precise mechanisms of action are unknown, it seems that in aquatic environment CH increases the pH level by releasing hydroxyl ions. Most of pathogenic bacteria cannot survive this environment. Also CH has some protein denaturizing effects that can facilitate the dissolution of pulp tissue [5-8]. CH needs a career material for obtaining proper consistency before being inserted into the canal. Normal saline, lidocaine and chlorhexidine are common materials for this purpose [9-13]. One of the most important criteria for selecting the carrier is maintaining the pH of CH mixture and its antimicrobial property [14-16]. 

Another aim of inter-appointment canal medication is to subside the periradicular inflammation and thus reducing the post-treatment pain [17]. Use of systemic or local corticosteroids for reducing inflammation and pain has always been appraised and numerous studies indicated that pain decrements after using corticosteroids [17-19]. 

To eliminate the side effects of systemic steroids, local intra canal medication with corticosteroids has gained interest in recent years. Ledermix is a combination of steroid/antibiotic which contains 1% triamcinolone and 3% demeclocycline. This formulation was first recommended for use in endodontics by Schroeder and Triadan in 1960 [20, 21]. Studies showed the ability of this medicine to penetrate into dentinal tubules and distribute in the periapical tissues [22-24]. Abbott *et al. *[25] indicated that slow releasing of active components of Ledermix and more stability of its properties amongst the advantages of this mixture. Constant pH of CH during mixing with Ledermix was showed by Taylor *et al. *[26]. Studies by Athanassiadis *et al. *[27, 28] showed that the antimicrobial property of CH during mixing with Ledermix remained unchanged, however deterioration of anti-inflammatory ability of Ledermix in mixture with CH may occur [29]. 

Because of the lack of studies in this regard, the aim of this *in vitro *study was to evaluate the effect of the mixing CH with betamethasone drop on the pH and antimicrobial ability of the mixture, using agar diffusion test and measuring the zone of inhibition (ZOI).

## Materials and Methods

Standard strain of *Enterococcus faecalis* (*E. faecalis*) (ATCC29212) was obtained from Microbiology Department, Shahid Sadoughi Medical School, Yazd, Iran. The 24-h blood agar culture was transferred to Tryptic soy broth (TSB) (Difco Laboratories, Detroit, Mich., USA) using a sterile loop to create a bacterial suspension with standard opacity equivalent to standard concentration of barium sulfate in a McFarland tube. CH powder (Pishro Dandan Co., Tehran, Iran) was placed on sterile glassy slap and was mixed either with saline or betamethasone drop (Abooreihan Co., Tehran, Iran) to make a paste with creamy consistency.

For agar diffusion test, 4 plates containing Mueller-Hinton broth (Difco Laboratories, Detroit, MI, USA) and *E. faecalis *culture media were prepared. In each plate, 5 holes (5 mm in diameter and 3 mm in depth) were created and the prepared bacterial media was placed into the holes (*n*=10). Two empty holes and two holes containing ampicillin disks were used as positive and negative controls, respectively.

All plates were placed in an incubator with 37^°^C temperature. After 24 h, the shortest distance from the outer margin of the wells to the initial point of bacterial growth was measured in mm as the ZOI. Finally, the obtained data were analyzed using the Mann-Whitney U test. For measuring the pH of both test mixtures, a digital pH meter was used.

## Results

The diameter of ZOI in positive control group was zero and in negative control group was 8 mm. The mean value of microbial ZOI was 3.4 mm and 3 mm, for CH+betamethasone and CH+saline samples, respectively. The difference between two groups was not statistically significant (*P*=0.143) (Table 1). Also the pH value was 12.5 in CH+saline group and 12.3 in CH+betamethasone group.

## Discussion

The present *in vitro* study showed that replacement of saline with betamethasone to prepare CH creamy paste, does not change its antibacterial activity on *E. faecalis*. 

Root canal cleaning is the main part of endodontic treatment; remaining bacteria in dentinal tubules can potentially cause treatment failure [4]. Cleaning of the dentinal tubules is not possible by mechanical instrumentation alone, so use of the intra-canal medicaments is recommended. CH is the most common material for this purpose [4].

The antimicrobial ability of CH is affected by the speed of its disintegration to calcium and hydroxyl ions, the latter elevating the environmental pH [4]. Hydroxyl ions are very active and immediately combine with lipids, proteins and nucleic acids and through peroxidation of lipids increase the permeability of bacterial membrane, denaturation of protein and inactivation of cellular enzymes [4]. This process, has a lethal effect on bacterial cells [30]. The optimal carrier for CH should not only preserve the alkaline pH but also should have no adverse effect on its disintegration rate nor distribution ability.

Considering the anti-inflammatory effect of corticosteroids, it can be assumed that if they could maintain the aforementioned optimal properties of CH, they can be a perfect carrier for CH paste by adding pain/inflammation reducing potential to the mixture[31].

The consistency of CH is an important factor during its placement in the canal. CH could be prepared in two thick and thin consistencies. Some studies do not consider the powder to liquid ratio important and mention mixing of powder and liquid until obtaining a thick or thin creamy mixture [32, 33]. By preparing creamy and more diluted mixture of CH, the disintegration speed of CH and its flow into the canal and dentinal tubules increase; so in this study the intra canal medicament was prepared in a creamy consistency [32].

Systemic or local corticosteroids are used for reducing the post endodontic pain. Commercial preparation of corticosteroid and antibiotic (Ledermix) is available for this purpose [22]. However, it is not available in Iran. In this study for the first time, mixture of CH and a corticosteroid drop (betamethasone) was used to take advantages of both antimicrobial activity of CH and anti-inflammatory effect of corticosteroids. In some previous investigations, mixture of CH and Ledermix has been assayed but not a single study has been published that employs the mixture of CH and a corticosteroid. The reason for selecting betamethasone in this study was the higher mucosal permeability of this drop [31]. 

**Table 1:** Mean (SD), of microbial zone of inhibition in mm

**Group (N)**	**Mean (SD) **	***P-*** **value**
**CH+saline (10)**	3 (0.3)	0.143
**CH+betamethasone (10)**	3.4 (0.5)	

According to the results, betamethasone preserved the appropriate alkaline pH of CH (~12.3). This value in normal saline group was 12.5. Taylor *et al.* [26] indicated that adding Ledermix to Pulpdent paste had no effect on its pH. Studies by Athanassiadis *et al.* [27, 28] also showed that the 50:50 mixture of CH and Ledermix had appropriate antimicrobial effect and Ledermix had no significant effect on antimicrobial property of CH. In the present study there was no significant difference in antimicrobial ability of CH mixture with betamethasone drop and normal saline, either. 

Abbott *et al. *[25], suggested the mixture of CH and Ledermix for long time intra-canal medication. However Athanassiadis *et al. *[29] showed that CH can destroy the triamcinolone acetonide content of Ledermix during 24 h and may decrease its anti-inflammatory effects. Based on this fact that inflammatory reaction and pain are maximal during first hours after endodontic treatment, this point has less clinical relevance [34]. 

The other important issue is avoidance of adverse chemical reaction between materials and the probably toxic byproducts. Taylor *et al. *[25] stated that combination of Ledermix and Pulpdent paste did not increase the toxicity of mixture on mammalian cells. The liquid form of betamethasone enables preparations with optimal consistency for inserting into the canal and this is one of the advantages of using betamethasone drop instead of Ledermix. Also use of this mixture prevents discoloration of the tooth; the phenomenon that is expected because of the demeclocycline content of Ledermix as shown by Kim and Day [35, 36].

## Conclusion

The mixture of calcium hydroxide with betamethasone drop had good antimicrobial effect against *Enterococcus faecalis*. Further studies are needed to confirm the value of this mixture in clinical settings.
